# Prevalence and risk factors of chronic kidney disease in urban adult Cameroonians according to three common estimators of the glomerular filtration rate: a cross-sectional study

**DOI:** 10.1186/s12882-015-0102-9

**Published:** 2015-07-07

**Authors:** Francois Folefack Kaze, Marie-Patrice Halle, Hermine Tchuendem Mopa, Gloria Ashuntantang, Hermine Fouda, Jeanne Ngogang, Andre-Pascal Kengne

**Affiliations:** Department of Internal Medicine and Specialties, Faculty of Medicine and Biomedical Sciences, University Teaching Hospital of Yaoundé, University of Yaoundé 1, Yaoundé, Cameroon; Department of Internal Medicine, Faculty of Medicine and Pharmaceutical Sciences, Douala General Hospital, University of Douala, Douala, Cameroon; Higher Institute of Health Sciences, Bangangté, Cameroon; Department of Internal Medicine and Specialties, Faculty of Medicine and Biomedical Sciences, Yaoundé General Hospital, University of Yaoundé 1, Yaoundé, Cameroon; Department of Internal Medicine and Specialties, Faculty of Medicine and Biomedical Sciences, Douala General Hospital, University of Yaoundé 1, Yaoundé, Cameroon; Department of Biochemistry and Physiologic Sciences, Faculty of Medicine and Biomedical Sciences, University of Yaoundé 1, Yaoundé, Cameroon; South African Medical Research Council, University of Cape Town, Cape Town, South Africa

## Abstract

**Background:**

Chronic kidney disease (CKD) is a major threat to the health of people of African ancestry. We assessed the prevalence and risk factors of CKD among adults in urban Cameroon.

**Methods:**

This was a cross-sectional study of two months duration (March to April 2013) conducted at the Cité des Palmiers health district in the Littoral region of Cameroon. A multistage cluster sampling approach was applied. Estimated glomerular filtration rate (eGFR) was based on the Cockcroft-Gault (CG), the four-variable Modification of Diet in Renal Disease (MDRD) study and Chronic Kidney Disease Epidemiology Collaboration (CKD-EPI) equations. Logistic regression models were used to investigate the predictors of CKD.

**Results:**

In the 500 participants with a mean age of 45.3 ± 13.2 years included, we observed a high prevalence of overweight and obesity (60.4 %), hypertension (38.6 %) and diabetes (2.8 %). The mean eGFR was 93.7 ± 24.9, 97.8 ± 24.9 and 99.2 ± 31.4 ml/min respectively with the MDRD, CG and CKD-EPI equations. The prevalence of albuminuria was 7.2 % while the prevalence of decreased GFR (eGFR < 60 ml/min) and CKD (any albuminuria and/or eGFR < 60 ml/min) was 4.4 and 11 % with MDRD, 5.4 and 14.2 % with CG, and 8.8 and 10 % with CKD-EPI. In age and sex adjusted logistic regression models, advanced age, known hypertension and diabetes mellitus, increasing body mass index and overweight/obesity were the predictors of albuminuria, decreased GFR and CKD according to various estimators.

**Conclusion:**

There is a high prevalence of CKD in urban adults Cameroonian, driven essentially by the commonest risk factors for CKD.

## Background

Sub-Saharan Africa (SSA) countries are undergoing demographic and epidemiological transition with the double burden of non-communicable and infectious diseases [1]. The adoption of western lifestyles, mostly in urban areas, contributes to increase the prevalence of hypertension and diabetes mellitus in this setting [2–4]. The above factors are associated with glomerular diseases and constitute the main etiological factors for chronic kidney disease (CKD) in SSA [5, 6]. CKD is emerging as one of the major health threats, affecting 10 % of adults worldwide and contributing every year to millions of premature deaths [7, 8]. Few studies have been conducted on CKD epidemiology in SSA [9]. These studies mostly of low methodological quality have revealed huge disparities in the prevalence of CKD across SSA regions depending on the definition, method for assessing glomerular filtration rate (GFR) and targeted population [4, 9–14].

In central Africa, previous studies have reported a high prevalence of CKD which affects young adults in their productive years; being higher in high risk groups such as people with hypertension, diabetes mellitus, obesity or HIV infection [10–12]. The present report presents findings from a study on the prevalence and risk factors of CKD in urban Cameroon.

## Methods

### Study setting and design

We carried a cross-sectional study of two months duration from March to April 2013 in all health areas of the Cité des Palmiers health district in the Littoral region of Cameroon. The Cité des Palmiers health district is the second largest and populous health district in Douala, the economic Capital of Cameroon. It comprises eight health areas with an estimated population of 423,253 inhabitants in 2012. The population is diversified representing the different ethnic and social groups in the country, and comprises students, traders, civil servants, housewives, and low, middle and high income earners from private sectors. This study was approved by the Cameroon National Ethics Committee.

We used a multistage cluster sampling to recruit 500 participants as Sumaili et al. in Kinshasa [11], corresponding to 62–63 subjects per health area. Sampling stages included the health area (first stage), the neighbourhood (second stage) and individuals (third stage). Adults were informed through community leaders, posters, leaflets and words of mouth, and requested to report to the chieftainship. All adults who reported on the day of recruitment benefited from a sensitization campaign, followed by a random selection of study participants and data collection.

### Data collection

Final year undergraduate medical students collected data between 8 a.m. to 12 a.m. for participants who provided a written informed consent. They used a pre-designed questionnaire to collect socio-demographic data (age, gender and occupation) and clinical information including personal history of existing conditions (hypertension, diabetes and gout), lifestyle characteristics (alcohol consumption and smoking), use of nephrotoxic agents (herbal medicines, foods addictive and street medicines), anthropometric measurements (weight, height and waist girth) and blood pressure variables. Blood pressure was measured according to the World Health Organization (WHO) guidelines [15] using an automated sphygmomanometer (OMRON HEM705CP, Omron Matsusaka Co, Matsusaka City, Mie-Ken, Japan) on the right arm with participants in a sitting position. All anthropometric measurements were performed three times and their average used in all analyses. In every participant, we drew 3 ml of whole blood from an antecubital vein into dry tubes for serum creatinine and collected mid-stream second morning urine for dipstick tests.

Dipstick tests were performed immediately after sample collection while blood specimens for serum creatinine were transported on ice-cooled containers to the biochemistry laboratory of the Douala General Hospital for processing. Urine dipstick tests were performed with CombiScreen 7SL PLUS 7 test strips (Analyticon Biotechnologies AG, D-35104 Lichentenfeis, Germany). Serum creatinine was measured with a kinetic modification of the Jaffé reaction using Human visual spectrophotometer (Human Gesellschaft, Biochemica und Diagnostica mbH, Wiesbaden, Germany) and Beckman creatinine analyzer (Beckman CX systems instruments, Anaheim, CA, USA). For any participant with positive dipstick [protein (at least traces), blood, leucocytes)], another urine sample was collected 2 to 3 weeks later to confirm the results. We excluded 11 (2.2 %) pregnant women and seven (1.4 %) participants with concomitant leucocyturia and urine nitrites.

### Definitions and calculations

Estimated glomerular filtration rate (eGFR, mL/min) used the Cockcroft-Gault (CG), the four-variable Modification of Diet in Renal Disease (MDRD) study and Chronic Kidney Disease Epidemiology Collaboration (CKD-EPI) equations [16–18]. CKD was defined by a confirmed positive dipstick albuminuria (at least traces) and/or eGFR < 60 ml/min/1.73 m^2^ according to K/DIGO guidelines which were used to stage participants for CKD [19]. eGFR classification was the following: G1 (eGFR ≥ 90); G2 (eGFR 60–89); G3a (eGFR 45–59); G3b (eGFR 30–44); G4 (eGFR 15–29) and G5 (eGFR < 15). Albuminuria was classified as: A1 (negative); A2 (trace to 1+) and A3 (at least 2+). Decreased GFR corresponded to any eGFR < 60 ml/min regardless the equation used. The following formula was used to convert serum creatinine from Jaffé reaction (SCr_Jaffé_) to standardized serum creatinine (SCr_Standardized_) to be used in MDRD and CKD-EPI formulas: SCr_Standardized_ = 0.95*SCr_Jaffé_ – 0.10 [20, 21]. Hypertension was defined as a systolic (SBP) ≥140 mmHg and/or a diastolic blood pressure (DBP) ≥90 mmHg or being on antihypertensive drugs. Diabetes was defined as self-reported history of doctor diagnosed condition or use of glucose control agents.

### Statistical analysis

Data analysis used SPSS® v.17 software for Windows® (SPSS Inc., Chicago, USA). We have reported the results as means and standard deviations, and counts and percentages. The Fisher exact test, Student *t*-test and Mann–Whitney *U* test were used to compare qualitative and quantitative variables across subgroups defined by sex, status for albuminuria, decreased GFR and CKD according to different GFR estimators. Age and sex adjusted logistic regression models were used to investigate the predictors of CKD. A *p*-value <0.05 was used to indicate statistically significant results.

## Results

### Baseline characteristics of the study population

The mean age was 45.3 years, similarly between men and women (*p* = 0.267), Table 1. Alcohol (68.9 vs. 37.8 %), tobacco use (12.7 vs. 0.4 %), and systolic blood pressure (137 vs. 128 mmHg) were higher in men compared to women (all *p* < 0.001). However, compared with men, women were more likely to have a history of hypertension (15.5 vs. 9.4 %, *p* = 0.041), to have high body mass index (BMI, 28.6 vs. 25.8 kg/m^2^, *p* < 0.001)), and to be overweight or obese (68.2 vs. 53.6 %, *p* = 0.001), Table 1. We observed a higher prevalence of CKD risk factors including hypertension (38.6 %), overweight and obesity (60.4 %), and longstanding use of herbal (57.8 %) and street (29.6 %) medicines in the study sample. As expected, serum creatinine was higher in men than women (*p* < 0.001) but women had a significantly higher mean estimated creatinine clearance by the CKD-EPI equation (103.5 vs. 95.5 ml/min, *p* = 0.005). Furthermore, estimated creatinine clearance was highest with CKD-EPI equation and lowest with the MDRD equation.

**Table 1:** Baseline characteristics, kidney function test and urine profile by sex

Characteristics		Overall	Men	Women	*P*
n (%)		500 (100)	267 (53.4)	233 (46.6)	-
Mean age, years (SD)		45.3 (13.2)	45.9 (13.5)	44.6 (12.8)	0.267
History of hypertension (%)		61 (12.2)	25 (9.4)	36 (15.5)	0.041
History of diabetes (%)		14 (2.8)	6 (2.2)	8 (3.4)	0.433
History of gout (%)		6 (1.2)	4 (1.5)	2 (0.9)	0.690
Tobacco use currently or formerly (%)		35 (7.0)	34 (12.7)	1 (0.4)	<0.001
Alcohol use currently or formerly (%)		272 (54.4)	184 (68.9)	88 (37.8)	<0.001
Longstanding use of herbal medicine (%)		289 (57.8)	160 (59.9)	129 (55.4)	0.303
Longstanding use of street medicine (%)		148 (29.6)	81 (30.3)	67 (28.8)	0.768
Mean SBP, mmHg (SD)		132 (24)	137 (23)	128 (24)	<0.001
Mean DBP, mmHg (SD)		81 (15)	82 (15)	81 (14)	0.267
Any hypertension (%)		193 (38.6)	109 (40.8)	84 (36.1)	0.311
Mean BMI, kg/m^2^ (SD)		27.1 (5.3)	25.8 (3.9)	28.6 (6.3)	<0.001
BMI > 25 (%)		302 (60.4)	143 (53.6)	159 (68.2)	0.001
Pulse (SD)		77 (13)	74 (13)	79 (12)	<0.001
Dipstick abnormalities (%)					
Albuminuria					0.542
A1		464 (92.8)	246 (92.1)	218 (93.6)	
A2		29 (5.8)	18 (6.7)	11 (4.7)	
A3		7 (1.4)	3 (0.6)	4 (0.8)	
Mean serum creatinine (jaffe), mg/dl (SD)		10.5 (2.6)	11.7 (2.6)	9.1 (1.8)	<0.001
Mean serum creatinine (standardized), mg/dl (SD)		9.9 (2.5)	11.0 (2.5)	8.6 (1.7)	<0.001
Mean Creatinine clearance, ml/min (SD)					
MDRD		93.7 (24.9)	93.8 (25.3)	93.7 (24.5)	0.981
CG		97.8 (24.9)	96.5 (24.8)	99.4 (24.9)	0.201
CKD-EPI		99.2 (31.4)	95.5 (28.6)	103.5 (33.9)	0.005
Stages of kidney function (eGFR), (%)					
MDRD	>90	259 (51.8)	139 (52.1)	120 (51.5)	0.983
	60–90	214 (42.8)	114 (42.7)	100 (42.9)	
	45–59	24 (4.8)	11 (4.1)	13 (5.6)	
	30–44	2 (0.4)	2 (0.7)	0 (0.0)	
	15–29	1 (0.2)	1 (0.3)	0 (0.0)	
CG	>90	296 (59.2)	155 (58.1)	141 (60.5)	0.854
	60–90	160 (32.0)	88 (33.0)	72 (30.9)	
	45–59	33 (6.6)	16 (6.0)	17 (7.3)	
	30–44	10 (2.0)	7 (2.6)	3 (1.3)	
	15–29	1 (0.2)	1 (0.4)	0 (0.0)	
CKD-EPI	>90	301 (60.2)	152 (56.9)	149 (63.9)	0.206
	60–90	177 (35.4)	104 (39.0)	73 (31.3)	
	45–59	19 (3.8)	8 (3.0)	11 (4.7)	
	30–44	2 (0.4)	2 (0.7)	0 (0.0)	
	15–29	1 (0.2)	1 (0.3)	0 (0.0)	

*A* albuminuria, *BMI* body mass index, *CG* Cockroft-Gault, *CKD-EPI* chronic kidney disease epidemiology collaboration, *DBP* diastolic blood pressure, *eGFR* estimated glomerular filtration rate, *MDRD* Modification of Diet in Renal Disease, *SBP* systolic blood pressure, *SD* standard deviation

### Staging of kidney function and prevalence of chronic kidney disease

The staging of kidney function according to various estimators is presented in Table 1. In general, there was no sex difference in the staging of kidney function, regardless of the estimator (all p > 0.206). None of the participants was in stage G5 regardless of the estimators used. Similar proportions of stages G3b and G4 were observed with CKD-EPI and MDRD estimators. The prevalence of albuminuria was 7.2 % while the prevalence of decreased GFR (eGFR < 60 ml/min) was 4.4, 5.4 and 8.8 % respectively based on GFR estimated from the CKD-EPI, MDRD and CG equations (Table 2). The prevalence of CKD (any albuminuria and/or eGFR < 60 ml/min) was 10.0, 11.0 and 14.2 % respectively for CKD-EPI, MDRD and CG equations (Table 3).

**Table 2:** Baseline characteristics by status for albuminuria and decreased GFR based on various kidney function estimators

Variables	Albuminuria			eGFR < 60 (MDRD)			eGFR < 60 (CG)			eGFR < 60 (CKD-EPI)		
	No	Yes	*P*	No	Yes	*P*	No	Yes	*p*	No	Yes	*p*
n (%)	464 (92.8)	36 (7.2)		473 (94.6)	27 (5.4)		456 (91.2)	44 (8.8)		478 (95.6)	22 (4.4)	
Sex (women)	218 (47.0)	15 (41.7)	0.605	220 (46.5)	13 (48.1)	>0.999	213 (46.7)	20 (45.5)	>0.999	222 (46.4)	11 (50.0)	0.828
Mean age, years (SD)	44.8 (13.0)	52.0 (14.1)	0.002	44.4 (12.7)	61.4 (10.9)	<0.001	43.6 (12.1)	62.9 (11.1)	<0.001	44.3 (12.6)	66.0 (9.1)	<0.001
History of HTA (%)	50 (10.8)	11 (30.6)	0.002	49 (10.4)	12 (44.4)	<0.001	48 (10.5)	13 (29.5)	0.001	49 (10.3)	12 (54.5)	<0.001
History of diabetes (%)	10 (2.2)	4 (11.1)	0.014	10 (2.1)	4 (14.8)	0.005	10 (2.2)	4 (9.1)	0.027	10 (2.1)	4 (18.2)	0.002
History of goutte (%)	4 (0.9)	2 (5.6)	0.063	6 (1.3)	0	>0.999	6 (1.3)	0	>0.999	6 (1.3)	0	>0.999
Tobacco use (%)	33 (7.1)	2 (5.6)	>0.999	34 (7.2)	1 (3.7)	0.711	34 (7.5)	1 (2.3)	0.348	35 (7.3)	0	0.389
Alcohol use (%)	255 (55.0)	17 (47.2)	0.390	259 (54.8)	13 (48.1)	0.554	253 (55.5)	19 (43.2)	0.153	262 (54.8)	10 (2.0)	0.512
Longstanding use of herbal medicine (%)	268 (57.8)	21 (58.3)	>0.999	271 (57.3)	18 (66.7)	0.424	264 (57.9)	25 (56.8)	>0.999	275 (57.5)	14 (63.6)	0.662
Longstanding use of street medicine (%)	133 (28.7)	15 (41.7)	0.128	143 (30.2)	5 (18.5)	0.278	135 (29.6)	31 (29.5)	>0.999	142 (29.7)	6 (27.3)	>0.999
Mean SBP, mmHg (SD)	132 (23)	138 (32)	0.199	132 (24)	142 (31)	0.033	131 (23)	144 (32)	0.001	132 (24)	145 (33)	0.011
Mean DBP, mmHg (SD)	81 (14)	85 (22)	0.094	81 (14)	86 (22)	0.074	81 (14)	84 (19)	0.168	81 (14)	86 (22)	0.136
Any hypertension (%)	174 (37.5)	19 (52.8)	0.077	174 (36.8)	19 (70.4)	0.001	166 (36.4)	27 (61.4)	0.002	176 (36.8)	17 (77.3)	<0.001
Mean BMI, kg/m^2^ (SD)	27.1 (5.3)	27.4 (5.8)	0.712	27.0 (5.2)	29.0 (7.0)	0.055	27.4 (5.3)	23.4 (3.5)	<0.001	27.0 (5.2)	28.5 (7.2)	0.200
BMI > =25 (%)	280 (60.3)	22 (61.1)	>0.999	284 (60.0)	18 (66.7)	0.549	287 (62.9)	15 (34.1)	<0.001	289 (60.5)	13 (59.1)	>0.999
Pulse (SD)	76 (12)	81 (14)	0.043	76.5 (12.6)	77.6 (14.9)	0.670	77 (13)	76 (14)	0.887	76 (13)	79 (16)	0.448

*BMI* body mass index, *CG* cockroft-Gault, *CKD-EPI* chronic kidney disease epidemiology collaboration, *DBP* diastolic blood pressure, *eGFR* estimated glomerular filtration rate, *MDRD* Modification of Diet in Renal Disease, *SBP* systolic blood pressure, *SD* standard deviation

**Table 3:** Baseline characteristics by status for chronic kidney disease based on various kidney function estimators

Variables	CKD (MDRD)			CKD (CKD-CG)			CKD (CKD-EPI)		
	No	Yes	*P*	No	Yes	*p*	No	Yes	*p*
n (%)	445 (89.0)	55 (11.0)		429 (85.8)	71 (14.2)		450 (90.0)	50 (10.0)	
Sex (women)	208 (46.7)	25 (45.5)	0.887	201 (46.9)	32 (46.6)	0.799	210 (46.7)	23 (46.0)	>0.999
Mean age, years (SD)	44.1 (12.7)	54.7 (13.4)	<0.001	43.4 (12.1)	56.9 (13.6)	<0.001	44.1 (12.4)	56.1 (14.2)	<0.001
History of hypertension (%)	44 (9.9)	17 (30.9)	<0.001	42 (9.8)	19 (26.8)	<0.001	44 (9.8)	17 (34.0)	<0.001
History of diabetes (%)	8 (1.8)	6 (10.9)	0.002	8 (1.9)	6 (8.5)	0.008	8 (1.8)	6 (12.0)	0.001
History of gout (%)	4 (0.9)	2 (3.6)	0.134	4 (0.9)	2 (2.8)	0.204	4 (0.9)	2 (4.0)	0.113
Tobacco use (%)	32 (7.2)	3 (5.5)	0.785	32 (7.5)	3 (4.2)	0.453	33 (7.3)	2 (4.0)	0.561
Alcohol use (%)	245 (55.1)	27 (49.1)	0.473	238 (55.5)	34 (47.9)	0.249	248 (55.1)	24 (48.0)	0.371
Longstanding use of herbal medicine (%)	256 (57.5)	33 (60.0)	0.774	249 (58.0)	40 (56.3)	0.797	260 (57.8)	29 (58.0)	>0.999
Longstanding use of street medicine (%)	130 (29.2)	18 (32.7)	0.639	124 (28.9)	24 (33.8)	0.403	129 (28.7)	19 (38.0)	0.192
Mean SBP, mmHg (SD)	132 (24)	138 (29)	0.081	131 (23)	140 (30)	0.008	132 (24)	139 (30)	0.052
Mean DBP, mmHg (SD)	81 (14)	85 (19)	0.076	81 (14)	94 (18)	0.138	81 (14)	84 (19)	0.127
Any hypertension (%)	161 (36.2)	32 (58.2)	0.002	154 (35.9)	39 (54.9)	0.004	163 (36.2)	30 (60.0)	0.002
Mean BMI, kg/m^2^ (SD)	27.0 (12.6)	28.2 (6.5)	0.107	27.4 (5.3)	25.2 (5.1)	0.002	27.0 (5.2)	27.9 (6.5)	0.268
BMI > =25 (%)	267 (60.0)	35 (63.6)	0.663	269 (62.7)	33 (46.5)	0.012	272 (60.4)	30 (60.0)	>0.999
Pulse (SD)	76 (13)	78 (14)	0.344	76 (13)	77 (13)	0.651	76 (13)	79 (14)	0.236

*BMI* body mass index, *CG* Cockroft-Gault, *CKD* chronic kidney disease, *CKD-EPI* chronic kidney disease epidemiology collaboration, *DBP* diastolic blood pressure, *MDRD* Modification of Diet in Renal Disease, *SBP* systolic blood pressure, *SD* standard deviation

### Correlates of albuminuria, decreased GFR and CKD

The distribution of baseline characteristics according to the presence of albuminuria, decreased GFR or CKD is shown in Tables 2 and 3. Advanced age and known hypertension and diabetes status were significantly associated with albuminuria, decreased GFR and CKD regardless the equation used meanwhile any hypertension was associated with decreased GFR and CKD. Decreased GFR and CKD estimated by CG were associated with increased BMI and overweight/obesity.

### Age and sex adjusted predictors of albuminuria, decreased GFR and CKD

Age and sex adjusted predictors of albuminuria, decreased GFR and CKD are presented in Tables 4 and 5 separately for each of the estimators. Advanced age was consistently and positively associated with all these outcomes, with the magnitude of the effects per year increase in age being 4 % (95%CI: 1–7 %) for albuminuria, 11 % (7–16 %) to 17 % (11–23 %) for decreased GFR, and 6 % (4–9 %) to 9 % (7–12 %) for CKD. With the exception of decreased GFR and CKD estimated by the CG estimator, known hypertension status was also significantly and positively associated with all the outcomes; meanwhile existing diabetes was borderline associated with albuminuria [OR 3.05 (95%CI: 0.98–12.8), *p* = 0.055], and CKD based on MDRD [3.22 (0.99–10.45), *p* = 0.051] and CKD-EPI [3.36 (1.02–11.07), *p* = 0.046] equations. Increasing BMI was significantly and negatively associated with CG-defined decreased GFR and CKD, and positively with MDRD and CKD-EPI defined decreased GFR, but not CKD. As a consequence, overweight/obesity was associated with lower odd of CG defined decreased GFR [0.11 (0.05–0.26), *p* < 0.001] and CKD [0.30 (0.17–0.54), *p* < 0.001]. The small number of outcomes precluded expanded multivariable regression analysis.

**Table 4:** Predictors of albuminuria and decreased GFR in age and sex adjusted logistic regressions

Variables	Albuminuria		eGFR < 60 (MDRD)		eGFR < 60 (CG)		eGFR < 60 (CKD-EPI)	
	OR (95 % CI)	*p*	OR (95 % CI)	*p*	OR (95 % CI)	*p*	OR (95 % CI)	*p*
Sex (women)	0.86 (0.43–1.72)	0.661	0.75 (0.33–1.75)	0.512	0.81 (0.39–1.69)	0.575	0.61 (0.23–1.64)	0.325
Age, per years	1.04 (1.01–1.07)	0.002	1.11 (1.07–1.16)	<0.001	1.16 (1.11–1.20)	<0.001	1.17 (1.11–1.23)	<0.001
History of hypertension	2.70 (1.18–6.17)	0.019	3.42 (1.41–8.28)	0.007	1.40 (0.61–3.22)	0.422	5.93 (2.06–17.05)	0.001
History of diabetes	3.05 (0.98–12.8)	0.055	2.59 (0.63–10.56)	0.184	1.02 (0.23–4.52)	0.974	2.88 (0.61–13.54)	0.180
History of gout	6.17 (1.07–35.61)	0.042	≅0	0.999	≅0	0.999	≅0	0.999
Tobacco use	0.70 (0.15–3.15)	0.642	0.65 (0.08–5.51)	0.695	0.33 (0.04–2.95)	0.324	≅0	0.998
Alcohol use	0.58 (0.28–1.22)	0.152	1.29 (0.51–3.26)	0.584	2.19 (0.96–4.96)	0.061	1.18 (0.39–3.59)	0.771
Longstanding use of herbal medicine	1.01 (0.50–2.03)	0970	1.67 (0.6904.03)	0.256	1.03 (0.50–2.15)	0.930	1.50 (0.54–4.15)	0.432
Longstanding use of street medicine	1.65 (0.82–3.33)	0.162	0.40 (0.14–1.15)	0.089	0.74 (0.34–1.64)	0.464	0.70 (0.24–2.06)	0.517
SBP, mmHg	1.00 (0.99–1.01)	0.894	1.01 (0.99–1.02)	0.881	1.00 (0.99–1.02)	0.675	1.00 (0.99–1.02)	0.640
DBP, mmHg	1.01 (0.99–1.03)	0.367	1.01 (0.98–1.03)	0.494	1.00 (0.97–1.02)	0.716	1.01 (0.98–1.04)	0.576
Any hypertension	1.28 (0.62–2.67)	0.506	1.85 (0.74–4.60)	0.185	0.97 (0.45–2.08)	0.942	2.48 (0.80–7.68)	0.116
BMI, per kg/m^2^	1.01 (0.94–1.08)	0.773	1.09 (1.01–1.18)	0.032	0.71 (0.62–0.81)	<0.001	1.10 (1.00–1.20)	0.055
BMI > =25	0.89 (0.43–1.83)	0.754	1.09 (0.43–2.74)	0.855	0.11 (0.05–0.26)	<0.001	0.72 (0.25–2.08)	0.544
Pulse	1.03 (1.00–1.05)	0.048	1.00 (0.97–1.04)	0.802	0.99 (0.96–1.02)	0.574	1.01 (0.98–1.05)	0.477

*BMI* body mass index, *CG* Cockroft-Gault, *CKD-EPI* chronic kidney disease epidemiology collaboration, *DBP* diastolic blood pressure, *eGFR* estimated glomerular filtration rate, *MDRD* Modification of Diet in Renal Disease, *SBP* systolic blood pressure, *SD* standard deviation

**Table 5:** Predictors of chronic kidney disease in age and sex adjusted logistic regressions

Variables	CKD (MDRD)		CKD (CG)		CKD (CKD-EPI)	
	OR (95 % CI)	*p*	OR (95 % CI)	*p*	OR (95 % CI)	*p*
Sex (women)	1.05 (0.59–1.89)	0.856	1.07 (0.62–1.87)	0.805	1.10 (0.59–2.04)	0.759
Age, per years	1.06 (1.04–1.09)	<0.001	1.09 (1.07–1.12)	<0.001	1.08 (1.05–1.10)	<0.001
History of HTA	2.40 (1.19–4.82)	0.014	1.61 (0.82–3.16)	0.164	2.66 (1.30–5.41)	0.007
History of diabetes	3.22 (0.99–10.45)	0.051	1.79 (0.53–5.99)	0.348	3.36 (1.02–11.07)	0.046
History of gout	3.95 (0.68–23.01)	0.126	3.02 (0.50–18.01)	0.226	4.62 (0.78–27.27)	0.091
Tobacco use	0.76 (0.21–2.70)	0.670	0.55 (0.15–2.00)	0.366	0.55 (0.12–2.48)	0.432
Alcohol use	0.65 (0.35–1.23)	0.188	0.56 (0.31–1.02)	0.060	0.63 (0.32–1.22)	0.171
Longstanding use of herbal medicine	1.12 (0.62–2.04)	0.699	0.94 (0.54–1.64)	0.838	1.03 (0.55–1.91)	0.934
Longstanding use of street medicine	1.05 (0.56–1.96)	0.879	1.09 (0.61–1.94)	0.778	1.38 (0.73–2.60)	0.325
SBP, mmHg	1.00 (0.99–1.01)	0.864	1.00 (0.99–1.01)	0.888	1.00 (0.99–1.01)	0.916
DBP, mmHg	1.00 (0.99–1.02)	0.616	1.00 (0.98–1.01)	0.720	1.00 (0.98–1.02)	0.849
Any hypertension	1.44 (0.77–2.66)	0.249	1.01 (0.57–1.80)	0.964	1.44 (0.75–2.76)	0.269
BMI, per kg/m^2^	1.04 (0.98–1.10)	0.170	0.88 (0.82–0.94)	<0.001	1.03 (0.97–1.09)	0.384
BMI > =25	0.92 (0.49–1.71)	0.797	0.30 (0.17–0.54)	<0.001	0.74 (0.39–1.42)	0.364
Pulse	1.01 (0.99–1.03)	0.440	1.00 (0.98–1.02)	0.872	1.01 (0.99–1.04)	0.308

*BMI* body mass index, *CG* Cockroft-Gault, *CKD* chronic kidney disease, *CKD-EPI* chronic kidney disease epidemiology collaboration, *DBP* diastolic blood pressure, *MDRD* Modification of Diet in Renal Disease, *SBP* systolic blood pressure, *SD* standard deviation

## Discussion

Our study assessed the prevalence and correlates of CKD in an urban adults Cameroonian population using the commonest estimators of kidney function. It revealed a high frequency of CKD and related risk factors in this population, with over one in ten participants having CKD regardless of whether CG, MDRD or CKD-EPI equations were used to estimate kidney function. This high prevalence of CKD appeared to be driven mostly by advanced age, hypertension, diabetes mellitus and adiposity.

Our study met the criteria of high quality applied in the meta-analysis by Stanifer et al., and revealed a higher prevalence of CKD in this setting, regardless the estimators used, in line with the findings of the meta-analysis and previous studies in Central and Western Africa region [9, 11, 14]. These results confirm the already suggested high burden of CKD in SSA setting. CKD prevalence rates in our setting approximate those reported in other low-to-middle income countries and implies that CKD is not affecting only high-income countries [22–25]. Furthermore, much higher prevalence rates have been reported in high risk groups such as hypertensive, diabetes, obese and HIV infected patients in SSA, inviting targeted and proactive screening of these patients [10]. However, lower prevalence rates have been observed in a country like Senegal, in spite of similar high frequency of CKD risk factors [4]. The discrepancy could be explained by the differences in CKD definition used as well as methods for assessing urinary albumin excretion and serum creatinine. The reported higher prevalence rate of CKD could be explained by the epidemiological transition; there is a dual burden of diseases in this setting characterized by the growing prevalence and the lower awareness, treatment adherence and control rates of non-communicable diseases, and the increase nephrotoxicity of drugs used in the treatment of communicable disease [1, 4, 11, 26–28].

Across estimators of GFR, the CG equation diagnosed more participants with decreased GFR and CKD while CKD-EPI and MDRD with ethnicity correction diagnosed about the same proportion of participants with both conditions, largely in line with existing and extensively discussed reports from previous studies [11, 13, 14]. The observed higher GFR estimated by CKD-EPI equation compared to others estimators could be related to the fact that this equation performed better than others especially at higher GFR [17, 18].

Regardless of the estimators used to assess CKD, advanced age, hypertension, diabetes mellitus and adiposity were the risk factors of CKD observed in this study as reported elsewhere [4, 11–13]. These are well known clinical and socio-demographic risk factors for CKD occurrence and progression to end stage renal disease (ESRD) [19]. Moreover, hypertension and diabetes mellitus are associated with glomerular diseases and constitute the main etiological factors for CKD in SSA [5, 6]. These findings invite appropriate management of such factors and an array of actions to tackle them as well as implementation of sensitization campaign to raise awareness, increase treatment adherence and improve control rate. This is important to reduce the growing prevalence of ESRD patients in this lower middle income country where social security programs are inexistent, and where patients with CKD are referred late to nephrologists [29, 30]. Furthermore, in Cameroon for instance, and in spite of government’s subsidies, patients with ESRD on renal replacement therapy must pay the equivalent of US$ 12 per dialysis session [US$ 1248 per annum, which is almost the gross national income per capita of US$ 1270 in 2013] in addition to the costs of caring for comorbidities [29–31].

### Strengths and limitations

The present study has some limitations including the semi-quantitative assessment of urinary albumin excretion using dipsticks, the non-validation of any of the equations used in SSA populations and the lack of three months control of positive findings to confirm the chronicity of renal injury as recommended by the KDIGO guidelines [19]. However, previous studies from Ghana and South Africa have found a high agreement between ethnicity corrected MDRD and CKD-EPI equations, supporting their use in this setting [13, 14]. Moreover, by conducting this study in only one urban health district of the country, there is little opportunity of assessing variations in the prevalence of CKD across the gradient of urbanization in the country. Lastly, the study was likely underpowered to reliably investigate the determinants of the disease. However, this study to our knowledge is the first to use a multistage cluster sampling to provide community-based data on the epidemiology of kidney disease in the country with the three estimators of kidney function. The inclusion of participants from a cosmopolite urban health district likely captures the diversity of the national population with our results likely reflecting the national urban prevalence of CKD.

## Conclusions

This study revealed that more than one in ten participants presented with CKD regardless the estimators used. This sizable prevalence of CKD, similar to those reported in developed countries, is driven essentially by the well-known clinical and socio-demographic risk factors for CKD. Actions are needed both to prevent further increase in the prevalence of CKD and to improve the detection and appropriate management of those with risk factors of the disease.

## Abbreviations

A: Albuminuria
BMI: Body mass index
CG: Cockroft-Gault
CKD: Chronic kidney disease
CKD-EPI: Chronic kidney disease epidemiology collaboration
DBP: Diastolic blood pressure
eGFR: Estimated glomerular filtration rate
MDRD: Modification of Diet in Renal Disease
SBP: Systolic blood pressure
SD: Standard deviation
